# Visa restrictions: A structural determinant of global health that must be confronted head-on

**DOI:** 10.1371/journal.pgph.0006178

**Published:** 2026-03-26

**Authors:** Luchuo Engelbert Bain, Samuel Akombeng Ojong, Julius Kirimi Sindi, Basile Njei, Lundi-Anne Omam, Catherine Kyobutungi

**Affiliations:** 1 African Population and Health Research Center (APHRC), Nairobi, Kenya; 2 Department of Psychology, Faculty of Humanities, University of Johannesburg, Johannesburg, South Africa; 3 Johns Hopkins Bloomberg School of Public Health, Johns Hopkins University, Baltimore, Maryland, United States of America; 4 UNICEF Mozambique, Maputo, Mozambique; 5 Section of Digestive Diseases, Department of Medicine, Yale University, New Haven, Connecticut, United States of America; 6 Engelhardt School of Global Health and Bioethics, Euclid University, Central African Republic; 7 Artificial Intelligence Programme, University of Cumbria, Carlisle, United Kingdom; 8 Ohio University Heritage College of Osteopathic Medicine, Athens, Ohio, United States of America; 9 Yale Liver Center, Yale New Haven Health, New Haven, Connecticut, United States of America; 10 Reach Out Cameroon, Bakweri Twon, Buea, Cameroon; 11 Cambridge Public Health Interdisciplinary Research Center, Department of Psychiatry, University of Cambridge, Cambridge, United Kingdom; PLOS: Public Library of Science, UNITED STATES OF AMERICA

## From “Visa Apartheid” to structural exposure

Visa restrictions can no longer be treated as a peripheral issue; they are a central concern in global health. In 2025 alone, thousands of researchers, public health officials, and clinicians from low- and middle-income countries (LMICs) were denied entry to high-income countries for conferences, training, and governance meetings, not for lack of credentials, but because they presented the wrong passport. These mobility constraints are not symmetrical as global health actors from high-income countries (HIC) rarely face comparable barriers when traveling to LMIC settings. Conversely, scholars and practitioners from LMICs routinely encounter what global health scholarship has long documented as “visa apartheid”, encompassing inequities in mobility, access, authorship, and epistemic authority; exclusions which have largely been framed as episodic injustices or ethical failures at the margins of an otherwise functional system [1–4].

The current wave of visa restrictions reveals a deeper structural problem: global health systems are built on assumptions of relatively unrestricted mobility that no longer hold. Although individual visa decisions and national policy shifts may be publicly visible and politically contested, their cumulative effects on who participates, who decides, and whose expertise travels remain insufficiently examined in global health governance debates [5,6]. Moreover, their harm profile seems to have shifted from the intermittent denial of access to structural weakening of institutions, particularly in low- and middle-income countries (LMICs) [7–9]. Viewed through this lens, visa regimens must be addressed at the systemic level given that they function as structural gatekeeping mechanisms that shape the global health ecosystem itself.

What is new is not exclusion itself, but its interaction with time-bound developmental processes central to health system performance. Meanwhile, this paradigmatic shift coincides with a convergence of forces reshaping the global health landscape: declining development assistance, restructuring of multilateral institutions, intensified calls for localization and country ownership, and growing geopolitical fragmentation [10–12]. Together, these dynamics expose the vulnerability and unsustainability of existing global health architectures that rely on cross-border mobility to ensure training pipelines, cross-country research and career progression, strengthen disease surveillance, and institutional participation in global governance.

## Visa regimes as structural determinants of global health

Visa regimes, defined here as state-governed systems of rules, practices, and administrative infrastructures that determine who may cross borders, for how long, and with what degree of scrutiny, are usually framed as matters of sovereignty, security, or migration control. They are not considered a health policy issue and remain largely absent from global health system design debates. Yet when examined through a systems lens, visa access functions as an invisible structural determinant of global health. They structure power, capacity, and credibility alongside financing, regulation, and governance architecture [11,13,14].

What distinguishes the current wave of visa restrictions imposed on scores of LMIC countries globally, is the scale and persistence of its mobility constraints, and how these interact with core system functions. The effects extend far beyond conference participation or short-term collaboration. Visa restrictions are disrupting how global health systems generate and sustain knowledge. Interrupted doctoral and postdoctoral training breaks mentorship chains, derails longitudinal research and weakens institutional memory. When training pathways depend on sustained mobility into high-income countries, visa barriers translate directly into fragmented and fragile research ecosystems [7,8].

Visa regimes also distort leadership pipelines within global health governance. Although visa restrictions are not the sole driver of these inequalities, they reinforce them by limiting the ability of many researchers, policymakers, and clinicians from low- and middle-income countries (LMICs) to participate consistently in international conferences, advisory groups, and governance forums where agendas are shaped and leadership pathways emerge. As a result, authority often concentrates among individuals whose passports or institutional affiliations allow routine international mobility [2,10,15]. Meanwhile, localization agendas decentralize implementation responsibilities without redistributing equivalent influence over decision-making. This produces an LMIC expertise–mobility conundrum, where technical expertise remains indispensable for delivery but structurally constrained from the global spaces where priorities and strategies are defined [11,12].

Also, visa restrictions represent a non-negligible feat of contradiction at the heart of localization, decentralizing implementation to countries, while authority, circulation, and legitimacy remain externally controlled. Ultimately, visa regimes silently arbitrate which institutions can realistically exercise ownership, directly contradicting stated commitments to equity and localization [3,10]. Seen through this lens, visa restrictions operate as durable structural determinants of global health system performance, not temporary administrative inconveniences.

## Re-architecting global health for mobility not against it

Visa restrictions simply expose or intensify documented harms and inequities in global health governance, rooted in historical, economic, and institutional asymmetries. Yet the field continues to respond to restricted mobility as an incidental anomaly, rather than a persistent structural constraint. Hence, conferences proceed even when many invited participants cannot obtain visas, governance meetings convene without those excluded, and major funders rarely confront the structural implications of these barriers [9]. Durable solutions require shifting incentives, governance arrangements, and institutional infrastructure across the ecosystem, including redesigning global health systems that no longer depend on mobility as a precondition for participation [6,11].

To this end, the global health ecosystem must:

### 1. Shift from individual mobility to institutional durability

Global health has over-invested in internationally mobile individuals while under-investing in institutional capacity in LMICs. Priority actions include strengthening institutional grant-holding, regulatory engagement, and agenda-setting authority within LMIC institutions, expanding regional doctoral and postdoctoral training pathways with shared supervision, and developing leadership models that do not depend on prolonged physical presence in high-income countries [10,7,8].

### 2. Build redundant, regional knowledge ecosystems

Dependence on a small number of global hubs creates systemic vulnerability. Regions should therefore be treated as knowledge ecosystems rather than peripheral sites. Investments should support regional centers for training, clinical trials, regulation, and policy development, alongside shared supervision, joint degrees, and rotating mentorship models that anchor capacity building locally and regionally. South–South collaboration should be prioritized as a primary mode of scientific exchange towards reducing over-reliance on any single institutional or geographic node [4,11,12].

### 3. Align localization rhetoric with mobility reality

Localization without mobility equity risks reinforcing dependency, hence the need for coordinated action across the ecosystem. LMIC governments should treat scientific capacity and researcher mobility as strategic national assets, while investing in domestic and regional postgraduate training and structured diaspora circulation [7,8]. Also, LMIC public health institutions should institutionalize shared platforms for ethics oversight, data governance, and regulatory science, reducing reliance on individual intermediaries [2,3]. HIC institutions must decentralise governance and authority and design partnerships that remain functional despite travel constraints [6,15]. Meanwhile, funders must prioritise investments in institutional depth, regional capacity, and redundancy, while channeling resources directly to LMIC institutions in line with ownership commitments [10,11].

## Conclusion

Visa regimes are not temporary political disruptions but structural features of a fragmented global order. The central question is therefore not only who bears the cost of mobility injustice, but whether global health systems are designed to function when mobility gets constrained. The geographic concentration of global health convenings and institutions far from the communities whose challenges they seek to address, and in contexts where key actors cannot participate due to visa barriers is no longer defensible; especially at a time when the world is calling for a new global health architecture. Without concrete changes in training, governance, and resource allocation, global health will remain constrained by mobility systems it has failed to confront. The time for confrontation is now.

## References

[1] Horton R. Offline: A global health crisis? No, something far worse. Lancet. 2020;395(10234):1410. doi: 10.1016/S0140-6736(20)31017-5 32359456 PMC7252047

[2] Abimbola S. The foreign gaze: authorship in academic global health. BMJ Glob Health. 2019;4(5):e002068. doi: 10.1136/bmjgh-2019-002068 31750005 PMC6830280

[3] Büyüm AM, Kenney C, Koris A, Mkumba L, Raveendran Y. Decolonising global health: if not now, when?. BMJ Glob Health. 2020;5(8). doi: 10.1136/bmjgh-2020-003394 PMC7409954 32759186

[4] Bandara S, Zeinali Z, Blandina DM, Ebrahimi OV, Essar MY, Senga J, et al. Imagining a future in global health without visa and passport inequities. PLOS Glob Public Health. 2023;3(8):e0002310. doi: 10.1371/journal.pgph.0002310 37611050 PMC10446228

[5] Hodson R. Science without borders. Nature. 2018;562(7727):S57. doi: 10.1038/d41586-018-06970-5 30333602

[6] Bandara S, Deng N, Pai M. The Global North is increasingly unsafe for global health meetings. Lancet. 2025;405(10491):1728–30. doi: 10.1016/S0140-6736(25)00757-3 40324446

[7] Kuhlmann E, Falkenbach M, Correia T, Humphries N, Hutchinson E, Rees GH, et al. Global health and care worker migration requires a global response. Health Policy. 2025;155:105305. doi: 10.1016/j.healthpol.2025.105305 40184861

[8] Aluttis C, Bishaw T, Frank MW. The workforce for health in a globalized context--global shortages and international migration. Glob Health Action. 2014;7:23611. doi: 10.3402/gha.v7.23611 24560265 PMC3926986

[9] Smith A, Persaud A, Bhugra D, Javed A, Liebrenz M. Restrictive visa policies harm global scientific exchanges. Lancet. 2024;403(10442):2376. doi: 10.1016/S0140-6736(24)00300-3 38823985

[10] Bump JB. Global health and its limitations: An historical perspective. Health Syst Reform. 2025;11(2):2478681. doi: 10.1080/23288604.2025.2478681 40202995

[11] Moon S, Røttingen J-A, Frenk J. Global public goods for health: weaknesses and opportunities in the global health system. Health Econ Policy Law. 2017;12(2):195–205. doi: 10.1017/S1744133116000451 28332461

[12] Gostin LO, Moon S, Meier BM. Reimagining Global Health Governance in the Age of COVID-19. Am J Public Health. 2020;110(11):1615–9. doi: 10.2105/AJPH.2020.305933 33026872 PMC7542258

[13] The Lancet Global Health. Passports and privilege: access denied. Lancet Glob Health. 2019;7(9):e1147. doi: 10.1016/S2214-109X(19)30337-7 31401989

[14] Marmot M. Social determinants of health inequalities. Lancet. 2005;365(9464):1099–104. doi: 10.1016/S0140-6736(05)71146-6 15781105

[15] Shiffman J, Smith S. Generation of political priority for global health initiatives: a framework and case study of maternal mortality. Lancet. 2007;370(9595):1370–9. doi: 10.1016/S0140-6736(07)61579-7 17933652

